# Intensity modulated radiotherapy in combination with endocrinotherapy in the treatment of middle and advanced Prostatic Cancer

**DOI:** 10.12669/pjms.35.5.591

**Published:** 2019

**Authors:** Sumei Zhang, Shufen Zhao, Xinzhen Fu

**Affiliations:** 1 Sumei Zhang, Department of Radiotherapy, Binzhou People’s Hospital, Shandong, 256610, China; 2 Shufen Zhao, Department of Oncology, Binzhou People’s Hospital, Shandong, 256610, China; 3 Xinzhen Fu, Department of Equipment, Binzhou People’s Hospital, Shandong, 256610, China

**Keywords:** Endocrinotherapy, Intensity modulated radiotherapy, Middle and advanced prostate cancer

## Abstract

**Objective:**

To evaluate the clinical efficacy of intensity modulated radiation therapy and endocrinotherapy for middle and advanced prostate cancer.

**Methods:**

Total 104 elderly patients with middle and advanced prostate cancer who were admitted to our hospital from November 2014 to August 2015 were selected using random number table method. They were divided into intensity-modulated radiotherapy combined with endocrinotherapy group (observation group) and conventional radiotherapy combined with endocrinotherapy group (control group), 52 each. The serum levels of prostate specific antigen (PSA) and free prostate antigen (f PSA) were measured three months after treatment. The short-term efficacy and toxic and side effects of the patients were observed, and the survival rate was recorded through three-year follow up.

**Results:**

The clinical effective rate of the observation group was 92.68%, and that of the control group was 70.73%; there was a significant difference between the two groups (P<0.05). The serum PSA and f PSA levels of the two groups were similar before treatment, but there was no significant difference (P>0.05). The serum PSA and f PSA levels after treatment were significantly lower than before treatment. The incidence of adverse reactions in the observation group was lower than that in the control group (P<0.05). The one-year and three-year survival rates of the two groups were significantly different (90.0 vs. 80.0%, 60.0 vs. 43.3%, P>0.05).

**Conclusion:**

Intensity modulated radiotherapy combined with endocrinotherapy was safe and well tolerated in the treatment of middle and advanced prostate cancer. It can improve the short-term efficacy and effectively reduce the serum oncological index concentration of patients. It can be promoted in clinics.

## INTRODUCTION

Prostate cancer is a common malignant tumor in male genitourinary system and one of the main causes of cancer related death in males in European and American countries.^1^ With the rapid development of China’s economy, the change of people’s living habits and the progress of population aging, the incidence of prostate cancer is increasing year by year, especially among the elderly over 70 years old, which has seriously affected the health of the elderly.^2^,^3^ The main causes of prostate cancer are related to genetic factors, sexual activities and eating habits.^4^

A study has shown that people with more sexual activities had an increased risk of developing prostate cancer,^5^ and a high-fat diet can also increase its incidence. The occurrence of prostate cancer is insidious, with no obvious symptoms or mild symptoms in the early stage, such as frequent urination, urgent urination, increased nocturia, thin and weak urinary stream etc. Most of the cases have been in the middle and late stage when obvious symptoms appear, and they are not suitable for surgical treatment because of their age, existence of multiple basic diseases and poor physical tolerance.^6^ Therefore, comprehensive treatment such as radiotherapy and endocrinotherapy has become common.^7^,^8^

The main role of radiotherapy is to destroy cancer cells, and endocrinotherapy aims at inhibiting the growth of cancer cells and the proliferation after radiotherapy. Intensity-modulated radiotherapy has been developed because of the limited efficacy and severe adverse reactions of the traditional radiotherapy. It is welcomed for its ability to increase target dose and reduce adverse reactions in normal tissues.^9^ For prostate cancer, endocrinotherapy or intensity-modulated radiotherapy alone has certain effect,^10^,^11^ but the clinical study of endocrinotherapy in combination with intensity-modulated radiotherapy in the treatment of prostate cancer is rarely reported in China. Based on this, intensity-modulated radiotherapy combined with endocrinotherapy was applied in the treatment of middle and advanced prostate cancer in this study, and its effect was compared with that of the conventional radiotherapy combined with endocrinotherapy.

## METHODS

One hundred and four elderly patients with middle and advanced prostate cancer who aged 65-85 years and were admitted to our hospital from November 2014 to August 2015 were included. They were divided into intensity-modulated radiotherapy combined with endocrinotherapy group (observation group) and conventional radiotherapy combined with endocrinotherapy group (control group) according to random number table. The fifty-two patients in the observation group were aged 65-85 years, with an average age of (75.37±6.29) years, and weighted 53-82 kg, with an average weight of (66.72±6.21) kg; there were 27 cases of TNM stage III and 25 cases of stage IV; there were 25 cases of pathological Gleason grade 4 and 27 cases of grade 5. The fifty-two patients in the control group were aged 65-83 years, with an average age of (76.19±6.92) years, and weighed 55-86 kg, with an average age of (67.88±5.62) kg; there were 28 cases of TNM stage III and 24 cases of stage IV; there were 23 cases of pathological Gleason grade 4 and 29 cases of grade 5. There was no significant difference in baseline data such as sex and age between the two groups (P>0.05). The therapeutic schedule of this study was reviewed and approved by the ethics committee of the hospital, and all patients signed informed consent before treatment.

### Inclusion criteria

included appearance of obvious symptoms such as dysuria, frequent urination, naked hematuria, hemospermia, perianal and urethral pain, urinary drip or urethra secretion, etc., hard nodules in rectal touch, TNM stage III or IV in RTE and MRI examination, prostatic cancer Gleason grade 4~5, invasion of cancer to prostatic capsule, levator ani muscle or external urethral sphincter, being diagnosed by histopathology and unsuitable for radical prostatectomy or refusal to accept surgical treatment, Gleason score between 8 and 10 points, and serum prostate specific antigen (PSA) ≥ 20 ng/mL.

### Exclusion criteria

included having diagnosed chronic prostatitis, benign prostatic hyperplasia and early prostate cancer, brain metastasis, infection, serious primary diseases in the heart, cerebral vessels, liver, kidney and hematopoietic system, malignant tumors from other sources, severe urinary tract infection, urethral stricture and bladder stones, other diseases which caused detrusor hyperactivity or detrusor amyotonia and urination disorder, and allergy to endocrinotherapy related drugs.

### Treatment methods

### Radiotherapy

Patients in the observation group received intensity-modulated radiotherapy. After computed tomography (CT) simulation orientation, scanning was performed on the area from L2 to 10 cm below the inferior margin of ischium, and the thickness of scanning layer was 5 mm. The gross tumor volume (GTV), including the whole prostate, bilateral seminal vesicle and clinical target, was determined according to CT images and magnetic resonance of pelvic cavity. Clinical target volume (CTV) was the same with GTV. The margin of planning target volume (PTV) was 1 cm away from the margin of CTV and then 0.5 cm. Then the plan of radiotherapy was formulated by physicist according to the individual condition of the patients. CTV was treated by 2.23 Gy each time, five times each week, for 35 times, and the total radiotherapy dose was 78.05 Gy. PTV was radiated by 2.17 Gy each time, five times each week, 35 times, and the total radiotherapy dose was 75.95 Gy. 95% of PTV should be given a dose no less than 76 Gy. There was dose limit for the surrounding sensitive organs, rectum and bladder V70 ≤ 25%, bilateral femur head V50 ≤ 5% and pubic bone V70 ≤ 25%. Patients in the control group received conventional radiotherapy. Radiotherapy was given in the front and back directions and bilaterally. The upper bound was the superior border of S1, the lower bound was the inferior border of ischial tuberosity. The lateral bound of the front and back fields and the upper and lower bounds of the lateral field was 1~2 cm outside the true pelvis. The prescribed dose was 2.0 Gy each time, once each day, five days each week, and the total dose was 70 Gy.

### Endocrinotherapy

Patients in the two groups were subcutaneously injected with 3.75 mg of leuprorelin (SFDA approval number: H20093852; Shanghai Lizhu Pharmaceutical Co., Ltd., China; specification: 3.75 mg) in the first day of radiotherapy, once every 28 days, and orally took 50 mg of bicalutamide (SFDA approval number: H20113535; Shanghai Fudan Fuhua Pharmaceutical Co., Ltd., China; specification: 50 mg), once each day. Drugs were withdrawal if the level of PSA was lower than 0.2 ng/mL and the lowest level was maintained for 2 months. If biochemical recurrence happened in the period of follow up, i.e., the level of PSA exceeded the lowest value, 2 ng/mL, the former medication continued.

### Observational indicators and evaluation of efficacy:

### Short-term efficacy

The focus was examined by CT or MRI in the 3^rd^ month after treatment. The efficacy was evaluated according to the Response Evaluation Criteria in Solid Tumors (RECIST).^12^ The efficacy was evaluated as complete remission (CR) if all the foci disappeared, as partial remission (PR) if the sum of the long diameter of the foci narrowed for more than 30%, as stable disease (SD) if the sum of the long diameter of the foci narrowed but has not reached PR or increased but not reached PD, and as progressive disease (PD) if the sum of the long diameter of the foci increased for more than 20% or new foci appeared. The computational formula of response rate (RR) was: CR + PR.

### Serum levels of PSA and f PSA

5 ml of peripheral blood was collected from each patient before treatment and in the 3^rd^ month after treatment. It was centrifuged at a centrifugal radius of 15 cm and 1000 r/min by a Japanese KOKUSAN H-103N centrifugal machine for 5 min. The concentration of PSA and f PSA in the serum sample was detected.

### Adverse reactions

Adverse reactions were evaluated according to the grading criteria of Radiation Therapy Oncology Group (RTOG).^13^

### Long-term efficacy

The one-year and three-year survival rates of the patients were evaluated in three-year follow up.

### Statistical analysis

Data was analyzed by SPSS ver. 21.0. Measurement data were expressed as mean±SD and processed by t-test. Enumeration data was expressed by number of cases (%) and processed by Chi-square test. Survival rate was compared using Kaplan Meier method. Difference was considered statistically significant if P<0.05.

## RESULTS

**Fig.1:**
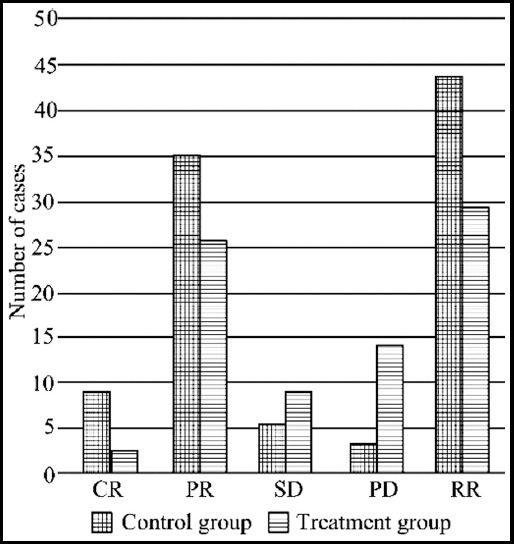
Comparison of short-term efficacy between the two groups

The overall response rate of the observation group was 84.6% (44/52), which was higher than that of the control group (55.8%; 29/52). The difference of the response rate between the two groups had statistical significance (X^2^=5.068, P<0.05, Fig.1).

The serum concentrations of PSA and f PSA of the two groups were close, and the difference was not statistically significant before treatment (P>0.05). The serum concentrations of PSA and f PSA of both groups decreased significantly after treatment, but the decrease amplitude of the observation group was significantly larger than that of the control group; the difference was statistically significant (P<0.05, Table-I).

**Table I:** Serum oncological indicators between the two groups before and after treatment.

Group	PSA		f PSA	

	Before treatment	After treatment	Before treatment	After treatment
Observation group	60.46±1.85	14.25±3.06*^#^	11.48±2.50	2.07±0.52*^#^
Control group	59.96±12.03	26.97±5.22*	11.67±2.47	3.56±0.85*

* ***Note:*** indicated P<0.05 compared to before treatment, ^#^ indicated P<0.05 compared to the control group.

The adverse reactions of the two groups included acute irritation signs of bladder such as urgent urination, frequent urination and painful urination, irritation signs of intestinal tract such as diarrhea, constipation and abdominal pain and bone marrow inhibition reactions such as leukocyte decrease, platelet decline and anemia. But most of them had mild symptoms, i.e., grade 1~2, which could be tolerated by the patients. Some patients with severe reactions had significantly improved symptoms after positive symptomatic treatment. All of them completed the treatment. The incidences of adverse reactions of the observation group and control group were 13.5% and 26.9% respectively, and the difference between the two groups had statistical significance (P<0.05, Table-II).

**Table II:** The comparison of adverse reactions between the two groups.

Group	Irritation signs of bladder	Irritation signs of intestinal tract	Grade 1 ~ 2 bone marrow suppression	Incidence of adverse reactions
Observation group	3(5.8)	1(1.9)	3(5.8)	7(13.5)
Control group	6(11.5)	3(5.8)	5(9.6)	14(26.9)
X^2^	/			4.379
P	/			<0.05

**Fig.2:**
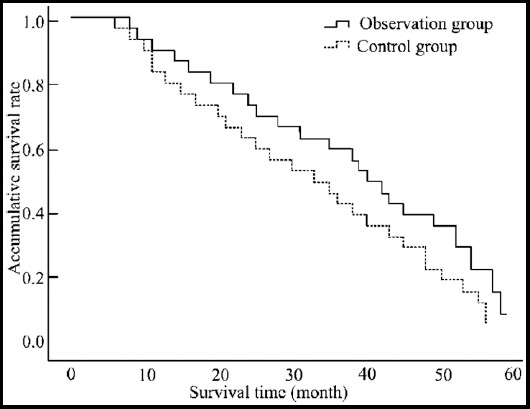
The comparison of survival curve between the two groups.

Up to August 2018, the 104 patients were followed up for 6~59 months. Survival time for more than 36 months was considered as censored data. The follow-up rate was 100%. The median survival time of the observation group was 40 months and 33 months respectively. The one-year and three-year survival rates of the observation group were 90.4% and 59.6% respectively, and those of the control group were 80.8% and 44.2% respectively. The Log-Rank test suggested that X^2^=2.048, and the difference between the two groups had no statistical significance (P>0.05). The comparison between the survival rates of the two groups is shown in Fig.2.

## DISCUSSION

Although the incidence of prostate cancer in China is lower than that in western developed countries, the number of patients with prostate cancer is huge because of the large population base and increasing age.^14^ Radical prostatectomy is commonly used in the treatment of early prostate cancer, and patients usually have a good prognosis. However, the elderly patients with middle and advanced prostate cancer often lose the chance of operation because of the relatively late stage and the limitation of their own complications.^15^ Combined treatment is usually advocated in the treatment of middle and advanced prostate cancer.^16^ The Guidelines of the Diagnosis and Treatment of Prostate Cancer updated by the European Urological Association clearly recommends the combination of radiotherapy and hormone therapy for the treatment of middle and advanced prostate cancer.^17^ Androgen dependence is the basis of endocrinotherapy for prostate cancer. Therefore, reducing androgen concentration in vivo and inhibiting the synthesis of adrenal-derived androgens are helpful to inhibit the conversion of testosterone to dihydrotestosterone and to block the binding of androgens and its receptors, so as to inhibit or control the growth of prostate cancer cells.^18^ In this study, patients received endocrinotherapy based on leuprorelin and bicalutamide. This regimen was effective in reducing the serum androgen level of patients, promote the death of androgen-sensitive cells in vivo, and achieve the purpose of suppressing tumor growth. It can alleviate the metastasis of tumors and eliminate the proliferation of cancer cells after radiotherapy, thus strengthening radiotherapy effect. Radiotherapy plays a role of radical, adjuvant or palliative treatment in the treatment of prostate cancer. Because the prostate is adjacent to the rectum, bladder and femoral head and other important tissues and organs, the radiation field will inevitably affect those tissues and organs, resulting in complications such as rectal ulcer bleeding, bladder perforation and femoral head necrosis, and its incidence is related to the radiation dose and illuminated volume, but the local control rate of tumor is also positively correlated with the dose of radiation.^19^ Conventional radiotherapy has been gradually replaced by intensity modulated radiotherapy because of its large irradiation field, the inevitable damage to important organs around tumors and the insufficient dose of radiation. Zelefsky et al. found that intensity modulated radiation therapy could reduce rectal and bladder radiation dose compared to the conventional chemotherapy.^20^,^21^ Deamaley et al. found that the serum PSA level in the intensity modulated radiotherapy group was lower than that in the conventional chemotherapy group^22^, and the time of biochemical recurrence and distant metastasis was longer than that in the conventional chemotherapy group. The results of the present study showed that the clinical response rate of the observation group was higher than that of the control group and the incidence of adverse reactions was significantly lower than that of the control group, suggesting that intensity modulated radiotherapy was superior to conventional chemotherapy in terms of efficacy and adverse reactions.

In order to further analyze the therapeutic effect of the two groups, the serum PSA and f PSA levels of the two groups were compared and analyzed. PSA as a glycoprotein secreted by the prostate acinar, is a serum tumor marker of prostate cancer with high sensitivity and specificity. Normally, the lymphatic system and the prostate acinar are isolated by the barrier. Once the tumor barrier is destroyed, a large number of PSA randomly enters the lymphatic system, resulting in increased concentrations of PSA and f PSA in blood.^23^ In this study, the serum PSA and f PSA levels in the two groups after treatment were significantly lower than those before treatment, but the intensity-modulated radiotherapy group decreased more significantly than the conventional group. It indicated that intensity-modulated radiotherapy combined with endocrinotherapy had a prominent role in regulating the serum oncological index concentration in patients with locally advanced prostate cancer, which is consistent with the report of Cao et al.^24^

In addition, the study also showed that the one-year and three-year survival rates of the observation group were higher than those of the control group, but there was no significant difference. The reason for insignificant difference might be that the combined therapy only increased the intensity of local treatment, but failed to reduce the growth of androgen-independent tumor cells in distant metastasis lesions, thus the long-term survival rate was not improved. It is also the limitation of local strengthening therapy. Although it can improve the short-term efficacy, it cannot be translated into survival benefits. It also suggested that lasting effect could be achieved when systemic therapy which could overcome androgen resistance was applied on the basis of improving local treatment intensity and endocrinotherapy.

## CONCLUSION

In conclusion, intensity-modulated radiotherapy combined with endocrinotherapy has favorable short-term effect and mild toxic and side reactions in the treatment of middle and advanced prostate cancer and it can effectively regulate the serum oncological index concentration. But in this study, the one-year and three-year accumulative survival rates of the two groups were close, which might be correlated to the short follow-up period and small sample size. The improvement effect of intensity-modulated radiotherapy on the long-term prognosis of patients with locally advanced prostate cancer remains to be verified by follow up in the future.

### Author`s Contribution

**SMZ:** Study design, data collection and analysis.

**SFZ & XZF:** Manuscript preparation, drafting and revising.

**SMZ & XZF:** Review and final approval of manuscript.

## References

[1] Sita TL, Petras KG, Wafford QE, Berendsen MA, Kruser TJ (2017). Radiotherapy for cranial and brain metastases from prostate cancer:a systematic review. J Neuro Oncol.

[2] Song LY (2012). The latest progress of diagnosis and treatment of prostatic cancer in China. Guide China Med.

[3] Xue L, Wang ZL, Li HC, Li Z, Chen Q, Zhang P (2017). RBPJ contributes to acquired docetaxel resistance in prostate cancer cells. Mol Cell Toxicol.

[4] Peek MC, Ahmed M, Napoli A, ten Haken B, McWilliams S, Usiskin SI (2015). Systematic review of high-intensity focused ultrasound ablation in the treatment of breast cancer. Br J Surg.

[5] Koneru H, Cyr R, Feng LR, Bae E, Danner MT, Ayoob M (2016). The impact of obesity on patient reported outcomes following stereotactic body radiation therapy for prostate cancer. Cureus.

[6] Son CH, Chennupati SK, Kunnavakkam R, Liauw SL (2015). The impact of hormonal therapy on sexual quality of life in men receiving intensity modulated radiation therapy for prostate cancer. Pract Radiat Oncol.

[7] Yagoda A, Petrylak D (2015). Cytotoxic chemotherapy for advanced hormone-resistant prostate cancer. Cancer.

[8] Cao F, Ramaseshan R, Cooper N, Elith CA, Nuraney N, Steiner P (2015). Template-based intensity-modulated radiation therapy:a cost-effective intensity-modulated radiation therapy planning procedure for prostate cancer. J Med Imag Radiat Sci.

[9] Son CH, Melotek JM, Liao C, Hubert G, Pelizzari CA, Eggener SE (2016). Bladder dose-volume parameters are associated with urinary incontinence after postoperative intensity modulated radiation therapy for prostate cancer. Pract Radiat Oncol.

[10] Yao H, Gong JL, Li L, Wu XF, Yu JJ (2012). Application of three dimensional conformal radiation therapy combined with high-intensity focused ultrasound for prostate cancer. Chin-Ger J Clin Oncol.

[11] Iizuka J, Hashimoto Y, Hashimoto Y, Kondo T, Takagi T, Nozaki T (2016). Efficacy and feasibility of intensity-modulated radiation therapy for prostate cancer in renal transplant recipients. Transplant Proc.

[12] Sage EK, Schmid TE, Geinitz H, Gehrmann M, Sedelmayr M, Duma MN (2017). Effects of definitive and salvage radiotherapy on the distribution of lymphocyte subpopulations in prostate cancer patients. Strahlenther Onkol.

[13] Yang X, Xu G, Lei Z, Song LP, Zhang Y (2012). Comparison of RECIST 1.1, RECIST 1.0 and WHO criteria for peripheral lung cancer response to treatment. Chin J Med Imag.

[14] Liu M, Shi XH, Yang F, Wang J, Xu Y, Wei D (2016). The Cumulative effect of gene-gene and gene-environment interactions on the risk of prostate cancer in Chinese men. Int J Environ Res Public Health.

[15] Hehemann MC, Baldea KG, Quek ML (2017). Prostate cancer in the elderly male:diagnostic and management considerations. Curr Geriatr Res.

[16] Hejazi J, Rastmanesh R, Taleban FA, Molana SH, Hejazi E, Ehtejab G (2016). Effect of curcumin supplementation during radiotherapy on oxidative status of patients with prostate cancer:a double blinded, randomized, placebo-controlled study. Nutrit Cancer.

[17] Thomsen FB, Roder MA, Rathenborg P, Brasso K, Borre M, Iversen P (2014). Enzalutamide treatment in patients with metastatic castration-resistant prostate cancer progressing after chemotherapy and abiraterone acetate. Scand J Urol.

[18] Chang JI, Bucci J (2016). Unusual side effect from a luteinizing hormone-releasing hormone agonist, leuprorelin, in the treatment of prostate cancer:a case report. J Med Case Rep.

[19] DiBiase SJ, Srivastav S (2016). The Influence of treatment energy on cardiac devices in patients undergoing intensity modulated radiation therapy (IMRT) for prostate cancer. Int J Radiat Oncol Biol Phys.

[20] Zheng J, Tao LS, Chen YS, Qin HB (2017). Clinical analysis of intensity modulated radiotherapy combined with endocrine therapy for 42 patients with advanced prostate cancer. Int J Urol Nephrol.

[21] Zelefsky MJ, Housman DM, Pei X, Alicikus Z, Magsanoc JM, Dauer LT (2012). Incidence of secondary cancer development after high-dose intensity-modulated radiotherapy and image-guided brachytherapy for the treatment of localized prostate cancer. Int J Radiat Oncol Biol Phys.

[22] Deamaley D, Hall E, Jackson C (2002). Phrase I trial of conformal radiotherapy following neoadjuvant hormone treatment in early prostate cancer. Int J Radiat Oncol Biol.

[23] Li G (2013). Application of radionuclide bone scintigraphy and serum indexes in diagnosis of bone metastasis in prostate cancer patients. J Hainan Med Coll.

[24] Cao JY, Wang QC, Wang Q, Wu G (2015). Evaluation of efficacy of I125 Brachytherapy combined with low-dose radiotherapy and hormontherapy in Treating intermediate and advanced prostate cancer. Int J Urol Nephrol.

